# Simultaneous Reverse Shoulder Arthroplasty and Open Reduction With Internal Fixation for Bilateral Proximal Humerus Fractures in the Elderly: A Report of Two Cases

**DOI:** 10.7759/cureus.28777

**Published:** 2022-09-04

**Authors:** Kai Sato, Taku Hatta, Kiyotsugu Shinagawa, Hiroshi Okuno, Shingo Nobuta

**Affiliations:** 1 Department of Orthopaedic Surgery, Tohoku Rosai Hospital, Sendai, JPN; 2 Department of Orthopedic Surgery, Joint Surgery, Sports Clinic Ishinomaki, Ishinomaki, JPN; 3 Department of Orthopaedic Surgery, Iwate Prefectural Central Hospital, Morioka, JPN

**Keywords:** total elbow arthroplasty, rheumatoid arthritis, reverse shoulder arthroplasty, open reduction with internal fixation, bilateral proximal humerus fracture

## Abstract

Simultaneous bilateral proximal humerus fractures (PHFs) caused by a single trauma are considered rare. We herein report two patients with bilateral PHFs treated based on our treatment strategy: reverse shoulder arthroplasty (RSA) to obtain rigid stability for one shoulder and open reduction with internal fixation (ORIF) to regain the mobility of external and internal rotation for the other. By using Neer’s four-segment fracture classification, patients underwent RSA for one shoulder with the more advanced fracture type and ORIF for the other. In Case 1, a 74-year-old female presented with bilaterally comminuted PHFs and underwent surgical treatment with RSA and ORIF. In Case 2, a 78-year-old female with the comorbidity of rheumatoid arthritis and a history of total elbow arthroplasty for both elbows had bilateral PHFs and underwent surgical treatment with RSA and ORIF. Postoperatively, both patients were instructed to perform exercises including passive range of motion (ROM) for a week, active ROM exercises for two weeks, and muscle strengthening for six weeks after the surgery. At the follow-up, both patients were satisfied with the pain relief and functional recovery. Especially, increased ROM for external and internal rotation was obtained in shoulders with ORIF. These case reports describe a viable treatment option comprising simultaneous RSA and ORIF, and this surgical approach may restore shoulder functions in cases of bilateral PHFs.

## Introduction

A proximal humerus fracture (PHF) is a common fracture in adult individuals, accounting for approximately 5%-8% of all adult fractures [1,2]. In particular, PHF has been recognized as the third-most common osteoporotic fracture, following proximal femur and distal radius fractures [3,4]. Displaced two-, three-, or four-bone fragment PHFs require surgical treatment to prevent residual pain and dysfunction of the shoulder, but the optimal surgical option among percutaneous pinning, open reduction, and internal fixation (ORIF) with intramedullary nailing or locking plate fixation and arthroplasties, such as hemiarthroplasty or reverse shoulder arthroplasty (RSA), remains controversial [5-7]. Of these, recent clinical studies show that RSA could be a reliable option for elderly patients with PHF to enable early mobilization exercises and to achieve functional recovery [6,7].

In contrast, simultaneous bilateral PHFs caused by a single trauma are considered rare. Although cases of the treatment of bilateral PHFs have been described [8-11], there is little consensus regarding valid treatment options for bilateral PHFs to achieve satisfactory outcomes. Determination of the treatment options for these complex injuries may be difficult since it remains unclear whether there are increased risks of adverse events, such as nonunion or malunion, due to a lack of intact upper extremities that would reduce the excessive stress during the healing process [8,9]. In addition, surgeons should note that poor outcomes can be associated with a delayed rehabilitation protocol following inadequate stability of the fragments. Among these case reports, a recent report demonstrated the efficacy of utilizing RSA bilaterally [8]. Although RSA can provide rigid stability to allow early immobilization, there is another concern that decreased mobility of external and/or internal rotation can remain postoperatively [12,13].

We herein report two patients with bilateral PHFs who were treated based on our treatment strategy: RSA to achieve rigid stability for one shoulder and ORIF to regain the mobility of external and internal rotation for the other. By using Neer’s four-segment fracture classification, patients underwent RSA for one shoulder with a more advanced fragment type, and ORIF was adopted for the other. Written informed consent has been obtained from the patients for the publication of their case report with any accompanying images.

## Case presentation

Case 1

**Figure 1:**
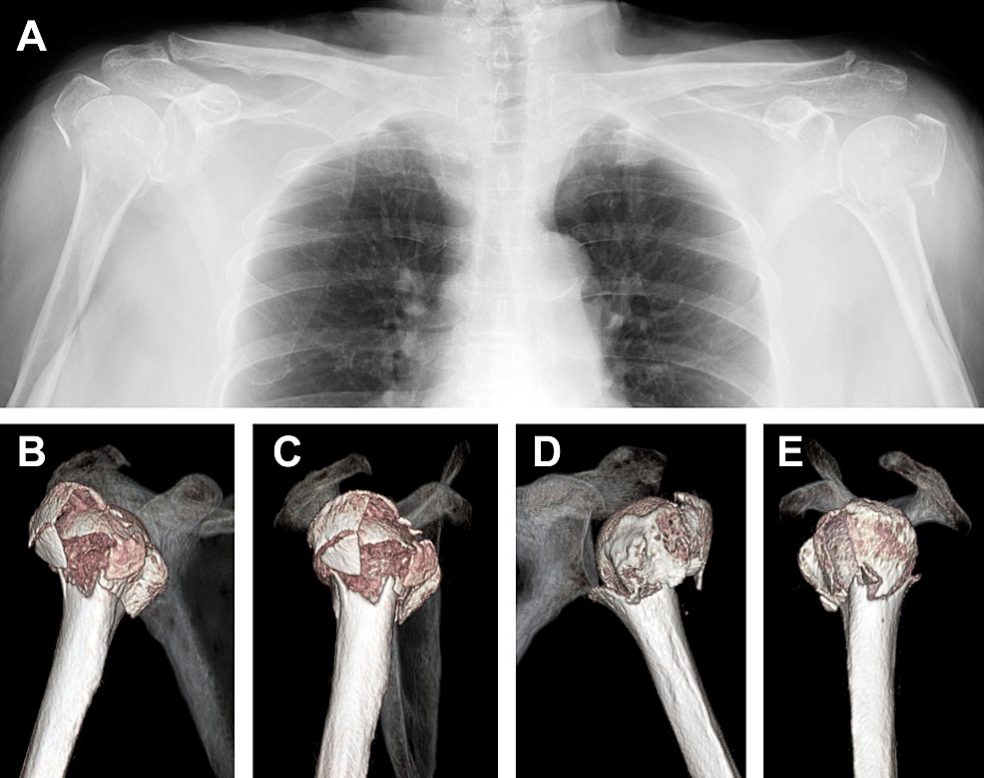
Case 1. Preoperative radiographic images The plain radiograph (A) and CT images (B, C: right, D, E: left) represent bilateral comminuted proximal humerus fractures.

A 74-year-old, right-handed female sustained bilateral PHFs by falling down a flight of stairs and placing both hands on the ground with the shoulders in forward flexion, the elbows in extension, and the wrist joints in dorsal flexion. Radiologically, the patient was diagnosed with bilateral comminuted PHFs: Neer four-part fracture of the right proximal humerus and Neer three-part fracture of the left proximal humerus (Figures 1A-1E).

MRI was not examined preoperatively since the patient had no history related to the shoulder symptoms. Six days after the injury, the patient underwent surgical treatment with RSA (combined use of the Trabecular Metal Reverse and Comprehensive; Zimmer Biomet Holdings, Inc., Warsaw, IN) for the right PHF and intramedullary nailing (T2 Proximal Humerus Nail; Stryker, Inc., Kalamazoo, MI) for the left PHF. Under general anesthesia, the patient underwent the surgery for both RSA and ORIF in the beach chair position.

**Figure 2:**
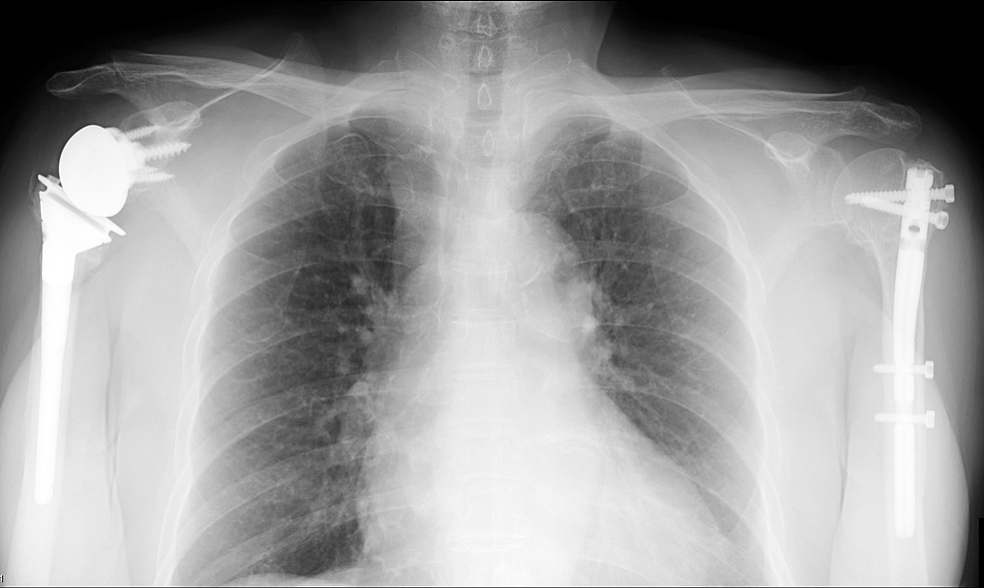
Case 1. Postoperative radiograph at the two-year follow-up

Postoperatively, both arms were immobilized in slings for a week, and she performed pendulum and passive range of motion (ROM) exercises for forward flexion under the tutelage of a therapist for her bilateral shoulders. The patient was instructed to perform active ROM exercises for forward flexion and external rotation at two weeks and muscle strengthening exercises at six weeks after the surgery. Since the patient did not receive any examination for osteoporosis, she was assessed with the dual-energy X-ray absorptiometry (DEXA) at one week after surgery, showing the bone mineral density (BMD) of the lumbar vertebrae (L2-L4) as 0.742 g/cm^2^ (T-score = -2.6 and Z-score = 1.3) and bilateral femoral necks as 0.572 g/cm^2^ (T-score = -2.0 and Z-score = 0.2). Based on the diagnosis of osteoporosis, the patient was treated with teriparatide for three months followed by denosumab. Radiographic examination revealed sufficient bony fusion without displacement at the left proximal humerus as well as at the tuberosities of the right proximal humerus (Figure 2).

**Figure 3:**
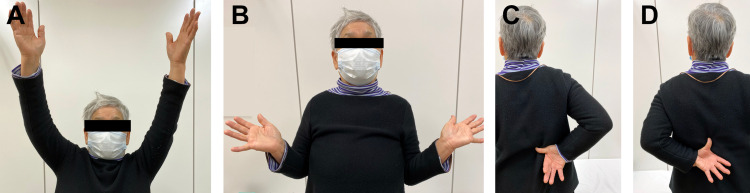
Case 1. Ranges of motion at the two-year follow-up Elevation (A, right: 120°, left: 135°) and external rotation (B, right: 15°, left: 45°) for bilateral shoulders. Internal rotation for right (C, the thumb at the 2nd lumber vertebra level) and left (D, the thumb at the 1st lumber vertebra level) shoulders.

At the two-year follow-up after surgery, the patient had no pain or restricted activities of daily living. The clinical outcomes assessed with the University of California-Los Angeles (UCLA) shoulder scale at the two-year follow-up showed 30 points for the right shoulder and 32 points for the left shoulder (Figures 3A-3D).

Case 2

**Figure 4:**
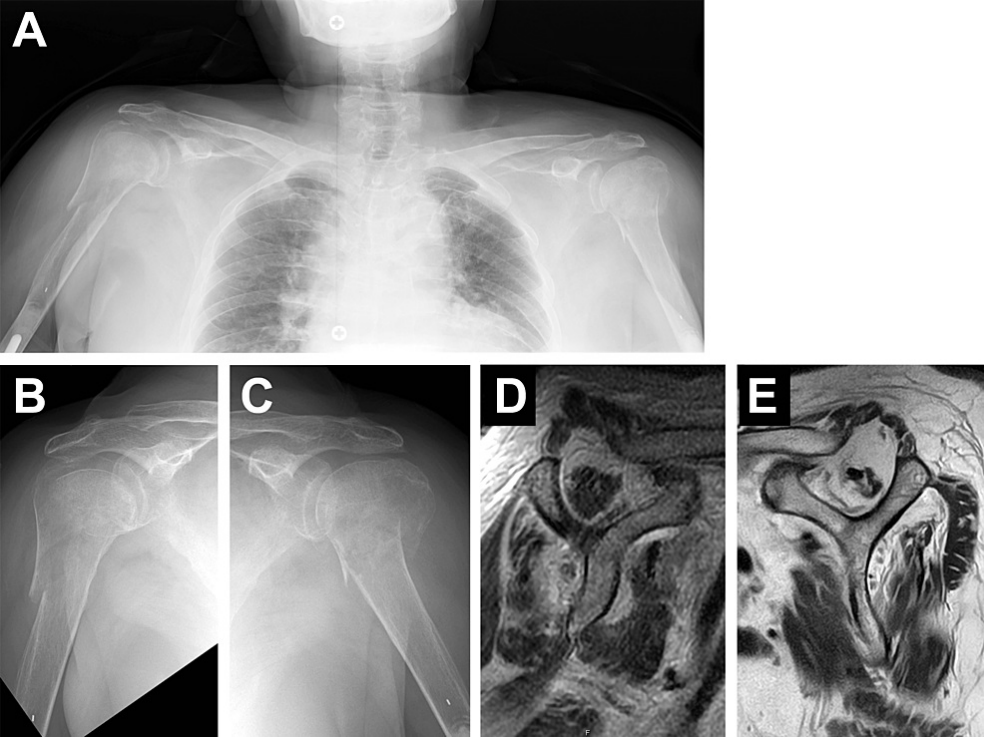
Case 2. Preoperative radiographic images The plain radiographs (A-C) show bilateral PHFs. The oblique sagittal T2-weighted images on MRI (D: right, E: left) represent muscle atrophy with/without fatty infiltration at the upper part of the subscapularis, supraspinatus, and infraspinatus muscles at bilateral shoulders.

A 78-year-old, right-handed female visited our clinic just after falling over a curb and placing both hands on the ground. The patient had comorbidity of rheumatoid arthritis treated with methotrexate and history of total elbow arthroplasty (TEA) for both elbows. In addition, the patient had received denosumab with a diagnosis of osteoporosis. At four months prior to the trauma, DEXA showed BMD of the lumber vertebrae (L2-L4) as 1.060 g/cm^2^ (T-score = 0.4 and Z-score = 2.2) and bilateral femoral necks as 0.595 g/cm^2^ (T-score = -1.8 and Z-score = 0.5). Radiographically, bilateral PHFs were identified; Neer three-part fracture on the right and Neer two-part fracture on the left (Figures 4A-4C). In addition, preoperative MRI showed widespread atrophy of the rotator cuff muscles with a continuity of the tendinocapsular structures in the right shoulder and a large rotator cuff tear with severe muscle atrophy and fatty infiltration in the left shoulder (Figures 4D-4E).

**Figure 5:**
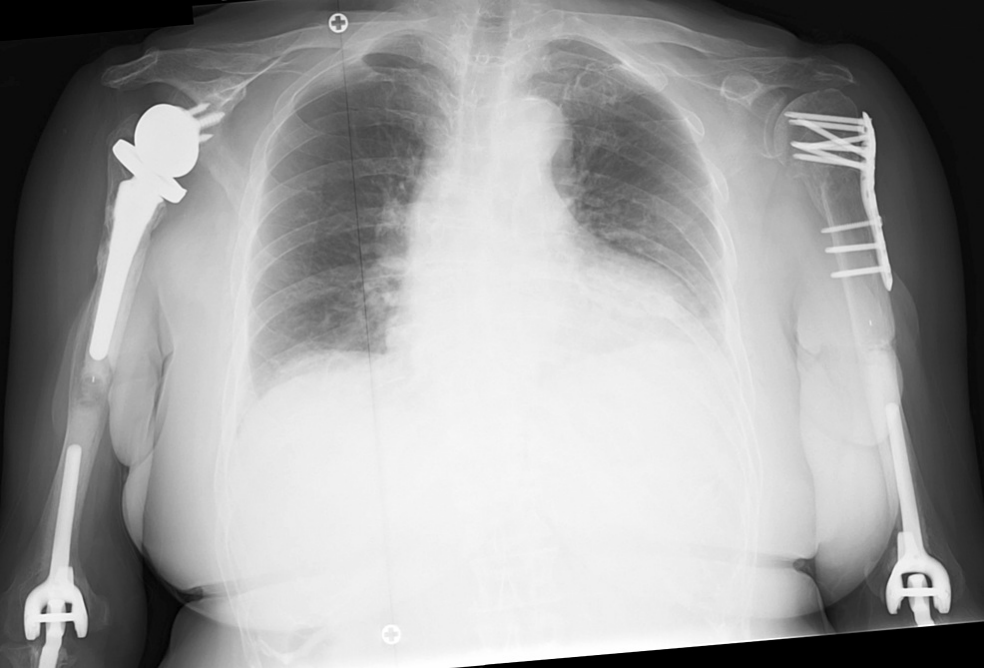
Case 2. Postoperative radiograph at the two-year follow-up

Nine days after the injury, the patient underwent surgical treatment with RSA (Comprehensive; Zimmer-Biomet) for the right PHF and with the locking plate fixation (PHILOS plate; Johnson & Johnson, Inc., New Brunswick, NJ) for the left PHF (Figure 5).

**Figure 6:**
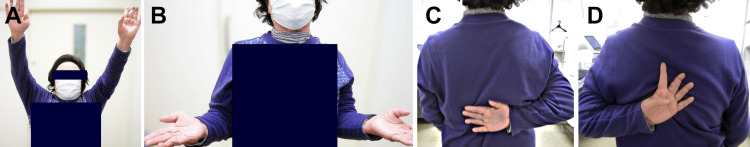
Case 2. Ranges of motion at the two-year follow-up Elevation (A, right: 125°, left: 120°) and external rotation (B, right: 15°, left: 25°) for bilateral shoulders. Internal rotation for right (C, the thumb at the 11th thoracic vertebra level) and left (D, the thumb at the 7th thoracic vertebra level) shoulders.

Postoperatively, bilateral arms were immobilized in slings for a week, followed by an instruction to perform exercises similar to those used in Case 1. One week after the surgery, the patient was treated with teriparatide for two months followed by denosumab. Although with slight pain around the left shoulder during moderate activities such as lifting the luggage at the one-year follow-up, the patient was satisfied with complete pain relief as well as functional recovery at the two-year follow-up, with a University of California-Los Angeles (UCLA) shoulder scale of 30 points for both shoulders (Figures 6A-6D).

## Discussion

We reported two cases of bilateral PHFs sustained simultaneously and treated with the surgical treatment comprising RSA for one shoulder with a more advanced fragment type according to Neer’s classification and ORIF for the other shoulder.

Several treatment options for bilateral PHFs have been introduced in case reports. Of these, surgical treatment with ORIF for bilateral PHFs has been found most frequently [10,14,15]. Maalouly et al.reported an elderly patient who underwent bilateral locking plate fixation for bilateral comminuted PHFs, which resulted in satisfactory outcomes; however, they mentioned that arthroplasties might be indicated, especially for osteoporotic, comminuted cases [10]. Ellanti et al. reported a patient who underwent surgical treatment with a combination of ORIF for the dominant shoulder and hemiarthroplasty for the contralateral shoulder [9]. They focused on comparing the postoperative functional recovery between the shoulders and concluded that a similar objective function was found after treatment with both options, although there was a subjective preference for the shoulder with ORIF, as it seemed more natural. Ceri et al. described an elderly patient treated with simultaneous bilateral RSAs for bilateral PHFs as an option to obtain an adequate function [8]. In contrast, Rodriguez-Corlay et al. reported a patient with bilateral comminuted PHFs who underwent conservative treatment without surgeries but achieved satisfactory outcomes at the short follow-up [11].

Although the optimal surgical option for the treatment of PHF remains controversial, recent studies have attempted to clarify the postoperative characteristics of each surgical option. A matched-pair analysis to compare ORIF and RSA for patients with PHF demonstrated that ORIF could result in better functional outcomes with increased Oxford Shoulder Scores and the Disabilities of the Arm, Shoulder, and Hand (DASH) scores than RSA [16]. A retrospective case-control study to compare ORIF, hemiarthroplasty, and RSA with a minimum of one-year follow-up showed no differences in functional recovery; whereas, a significantly decreased revision rate was found in patients who underwent RSA [17]. Several studies have also indicated RSA as a preferred surgical option for decreased revision rate [12,13]. On the other hand, these mentioned postoperative ROMs in shoulders with RSA: regained forward elevation but decreased external [12] or internal rotation [13]. Most recently, a systematic review to compare clinical outcomes among surgical options suggested that ORIF could achieve the highest improvement in postoperative QOL and pain relief; whereas, RSA could provide satisfactory outcomes with a minimal revision rate [18].

For our cases, we decided to perform surgery with the following treatment strategy: RSA for a shoulder with a more advanced type of displaced fragments according to Neer’s classification and ORIF for the other shoulder. By selecting these options for patients with PHFs, we expect that RSA could provide early functional recovery with mechanically rigid stability against the loadings, and ORIF could achieve wide ROMs (i.e., external and internal rotation) and QOL improvement with anatomically reduced structures. Although there have been no reports applying the present approaches to bilateral PHFs, this case report may support our treatment strategy as a valid option for restoring the function of bilateral shoulders.

No reports have described the treatment of bilateral PHFs in patients with rheumatoid arthritis who have undergone bilateral TEA, as shown in Case 2. A recent study found rheumatoid arthritis to be an independent risk factor for nonunion following conservative treatment of proximal PHFs [19]. In Case 2, preoperative MRI showed her bilateral rotator cuff pathologies; especially, a large rotator cuff tear with chronic alteration (severe fatty degeneration and muscle atrophy) was identified in the left shoulder, which had the PHF with less displaced fragments. Prioritizing the amount of displacement fragments over the rotator cuff pathologies, we determined to perform RSA for the right PHF and ORIF for the left PHF. Although Case 2 achieved pain relief and a satisfactory functional recovery in the bilateral shoulders with the current treatment option comprising ORIF and RSA, further postoperative follow-up will be required to assess whether or not the current surgical option can be preferred in such individuals. Of particular note, the potential risk of humerus fracture between the total elbow and shoulder arthroplasties should be considered [20].

## Conclusions

These two case reports described a treatment option comprising simultaneous RSA and ORIF for a rare injury of bilateral PHFs. We described our treatment strategy: RSA for one shoulder with more displaced fragments to achieve early functional recovery with mechanically rigid and tolerable structures, and ORIF for the other to provide wider ROMs with an anatomically reduced condition. The clinical outcomes of the current cases may indicate this surgical technique as a viable option to restore function in cases with bilateral PHFs.
